# Clustergrammer, a web-based heatmap visualization and analysis tool for high-dimensional biological data

**DOI:** 10.1038/sdata.2017.151

**Published:** 2017-10-10

**Authors:** Nicolas F. Fernandez, Gregory W. Gundersen, Adeeb Rahman, Mark L. Grimes, Klarisa Rikova, Peter Hornbeck, Avi Ma’ayan

**Affiliations:** 1 Department of Pharmacological Sciences, Mount Sinai Center for Bioinformatics, BD2K-LINCS Data Coordination and Integration Center (DCIC), Icahn School of Medicine at Mount Sinai, New York, New York 10029, USA; 2 Human Immune Monitoring Core, Icahn School of Medicine at Mount Sinai, New York, New York 10029, USA; 3 Center for Structural and Functional Neuroscience, University of Montana, Missoula, Montana 59812, USA; 4 Cell Signaling Technology Inc., Danvers, Massachusetts 01923, USA

**Keywords:** Functional clustering, Research data, Software

## Abstract

Most tools developed to visualize hierarchically clustered heatmaps generate static images. Clustergrammer is a web-based visualization tool with interactive features such as: zooming, panning, filtering, reordering, sharing, performing enrichment analysis, and providing dynamic gene annotations. Clustergrammer can be used to generate shareable interactive visualizations by uploading a data table to a web-site, or by embedding Clustergrammer in Jupyter Notebooks. The Clustergrammer core libraries can also be used as a toolkit by developers to generate visualizations within their own applications. Clustergrammer is demonstrated using gene expression data from the cancer cell line encyclopedia (CCLE), original post-translational modification data collected from lung cancer cells lines by a mass spectrometry approach, and original cytometry by time of flight (CyTOF) single-cell proteomics data from blood. Clustergrammer enables producing interactive web based visualizations for the analysis of diverse biological data.

## Introduction

The diversity of high content experimental methods in biomedical research is rapidly growing. Despite the accelerating pace of data acquisition, our ability to effectively generate insights from this data is lagging behind. Data visualization is a central tool for the initial analysis of biological data, and dimensionality reduction techniques, such as principal component analysis (PCA)^1^ and t-distributed stochastic neighbor embedding (t-SNE)^2^ are commonly employed to project high dimensional data onto two or three dimensions so it can be visualized. However, the transition from a high dimensional to a low dimensional space is costly, often resulting in loss of information. A clustergram, or a heatmap, on the other hand, is one of several techniques that directly visualizes data without the need for dimensionality reduction^3^. Clustergrams are easy to interpret and are widely used to visualize biological data in print publications. Hierarchically clustered heatmaps can also be used to visualize biological networks by displaying network connections in a symmetric adjacency matrix^4^. In such a display, the nodes of the network are the rows and columns and network links are represented as the cells within the matrix.

**Figure 1:**
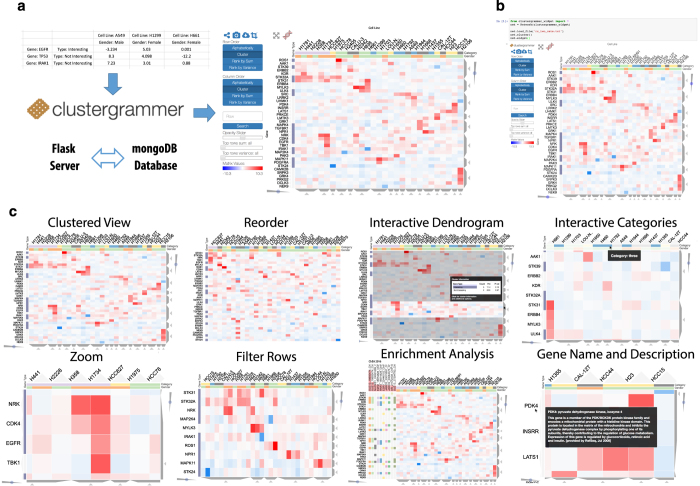
Clustergrammer web app, Jupyter widget, and interactivity. (**a**) Users can generate interactive and shareable heatmap visualizations using the Clustergrammer web application by uploading a matrix file at the homepage where they are redirected to a permanent and shareable visualization of the data. User data is clustered on the server side using default parameters. The visualization page includes three views of the data: a clustered heatmap, a similarity matrix heatmap of the columns, and a similarity matrix heatmap of the rows (not shown). (**b**) The Clustergrammer-widget can be used within a shareable Jupyter Notebook to produce interactive visualizations alongside code and markup text. (**c**) Clustergrammer implements many interactive features to enable intuitive data exploration including: zooming, panning, reordering, row filtering, interactive dendrograms, interactive categories, gene name/description lookup, and enrichment analysis.

**Figure 2:**
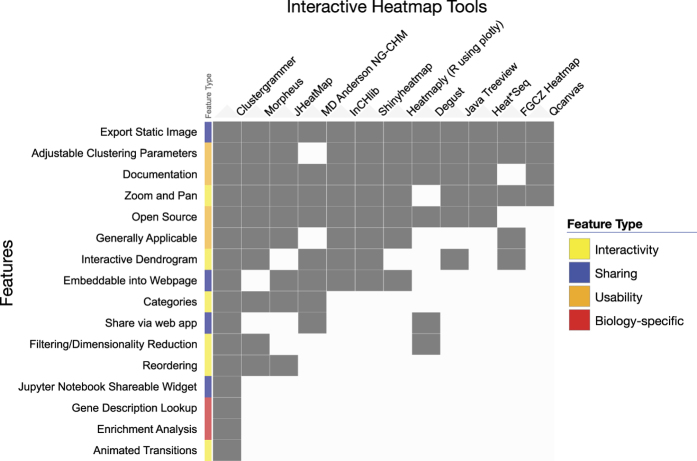
Interactive Heatmap Tool Feature Comparison. The heatmap compares interactive heatmap tools (shown as columns) based on their available features (shown as rows). The table was created using Clustergrammer where rows and columns are sorted by sum. Feature-categories are encoded using four colors. The interactive version can be found at https://maayanlab.github.io/interactive_heatmap_features/.

While there are several desktop software tools that are capable of producing interactive clustergrams^5–8^, these tools require software installation, and are only capable of exporting static images for sharing. Interactivity can only be shared if the end user also has specialized software installed. Improvements in browser capabilities and web-based visualization technologies such the JavaScript library data driven documents D3.js^9^ have led to the development of advanced web-based applications with animated and interactive features. There are several interactive web-based tools that can produce clustered heat maps^10–21^. However, most of these tools have limited interactivity, sharing capabilities, for example, by embedding them within web applications such as Jupyter Notebooks, as well as native integration with biology specific analysis methods such as enrichment analysis. Several tools that were developed to generate interactive heatmaps^16,18,20,21^ are built on top of existing visualization tools. For example, the Plotly interactive heatmap feature is hosted using the RShiny client/server architecture which lacks a database. This limits customizability and sharing capabilities. Clustergrammer is a custom built data visualization tool that is developed using the D3.js JavaScript library for the front-end, and Python for the back end. Clustergrammer generates shareable interactive visualizations via a web application, or as a Jupyter Notebook widget (Fig. 1). Clustergrammer offers unique features when compared with existing interactive heatmap tools^10–21^ (Fig. 2).

## Results

### The clustergrammer web application and jupyter notebook widget

The Clustergrammer web application provides the ability to generate shareable interactive visualizations by uploading a tab-separated data matrix file (Fig. 1a). Once this file is uploaded, the user is redirected to a permanent and shareable URL that contains the Clustergrammer generated interactive heatmap. By default, the page contains three views: the clustered heatmap, a similarity matrix heatmap of the columns, and a similarity matrix heatmap of the rows. The Clustergrammer web application can also be accessed programmatically with a RESTful application programming interface (API). The Clustergrammer web application is built using Python with the Flask library connected to a MongoDB database (Fig. 1a).

Clustergrammer visualizations can alternatively be embedded within Jupyter Notebooks^22^ as interactive widgets. The Clustergrammer-Widget enables generating interactive heatmap visualizations in context of text, code, and other analyses. Jupyter notebooks with embedded interactive heatmaps can be shared on the web using GitHub and the notebook rendering service, NBviewer (Fig. 1b), Clustergrammer visualizations embedded within Jupyter Notebooks are portable and can be integrated into existing workflows. Several case studies that utilize the Clustergrammer-Widget within a Jupyter Notebook have been developed for demonstration (see https://clustergrammer.readthedocs.io/case_studies.html). The Clustergrammer core libraries, Clustergrammer-JS and Clustergrammer-PY, can also be utilized as a tool-kit to generate interactive visualizations by developers for their own applications. The Clustergrammer web application and the Jupyter Widget utilize the same core libraries, Clustergrammer-JS and Clustergrammer-PY, and hence most, but not all, features that are available in the core libraries, are also available in the web application and Jupyter Widget. One important difference of a feature only available in Clustergrammer-PY and Clustergrammer-Widget, but not in the web application, is the ability to set category colors.

### Interactive visualization

Clustergrammer has interactive features including: zooming, panning, searching, selecting, and reordering with animated transitions (Fig. 1c). Moving the mouse over tiles on the heatmap or row/column labels display additional information as tooltips (Fig. 1c). The Clustergrammer sidebar contains controls for interacting with the visualization by sharing the link of the visualization page, taking a snapshot, downloading the data, cropping a section, row and column reordering, row search, opacity control, and row filtering (Fig. 1c). The sidebar buttons allow users to reorder rows and columns by sum, variance, hierarchical clustering, or by label. Users can reorder single row or column by double-clicking its title, or groups of rows and columns by clicking on the category title. For small matrices, reordering events are animated to help visually track the transformation. Clustergrammer enables users to interactively perform dimensionality reduction by filtering rows based on sum or variance using the sidebar row filter sliders (Fig. 1c). Clustergrammer immediately updates the clustering and animates the transitions to help users track the transition. This feature can be useful for filtering out parts of data based on interest, for example, rows with low variance.

Row and column dendrogram trees are typically used to show the results of hierarchical clustering. Clustergrammer visualizes the same information by displaying a single slice of a dendrogram using trapezoids (Fig. 1c). Sliders can be used to toggle between slices of the dendrogram tree to move across the different levels. Clicking or mouse-hovering over a dendrogram trapezoid brings up information about the cluster, and clicking on the trapezoid enables exporting the cluster. Dendrogram information includes a breakdown of the categories present in the cluster, as well as the enrichment *P*-values, calculated using the Binomial proportion test, for each category in the selected cluster. This feature can be useful for determining how prior knowledge categorization compares to data driven clustering, and whether a cluster is enriched for a specific category. Clicking on the dendrogram-crop-buttons filters the visualization to show only the selected cluster.

Clustergrammer implements several systems-biology specific features that facilitate the analysis of gene- and protein-level biological data (Fig. 1c). To utilize these features, row names must be official mammalian Entrez gene symbols. To streamline the process of looking up the description of each gene, Clustergrammer automatically displays the full name and description of a gene as a tooltip when a user moves the mouse over a gene name. Gene full name and description are obtained via the Harmonizome API and reflect the most up to date version of this information^23^. Another common function when exploring clusters from transcriptomics and proteomics studies is gene set enrichment analysis^24^. Clustergrammer integrates enrichment analysis features using the Enrichr API^25^. Clusters of genes and proteins can be exported to Enrichr using the interactive dendrogram. Enrichment analysis results can also be imported and visualized directly in Clustergrammer. By clicking the Enrichr logo at the top left of the interface, users can select a gene set library from Enrichr to query. The enrichment results are displayed using a bar chart and row-categories. This functionality can be used to associate enrichment results with specific genes. Users can also perform enrichment analysis on specific clusters of genes by first cropping the matrix using the dendrogram crop buttons or the brush crop feature.

### Case study I: Visualization of lung cancer post-translational modification data

**Figure 3:**
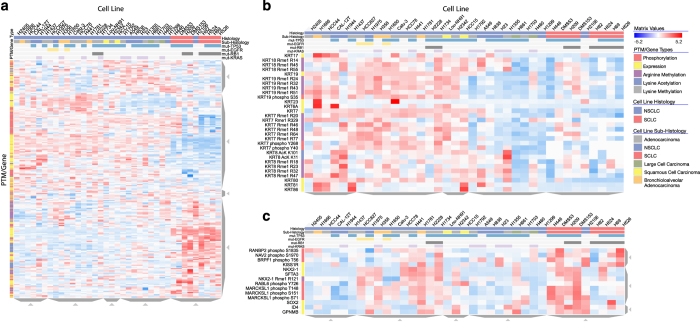
Lung cancer post-translational modification and gene expression regulation. (**a**) Lung cancer cell lines (columns) were clustered based on a combination of PTMs and mRNA expression data (rows). (**b**) Zooming into a cluster containing Keratins with commonly up-regulated expression and post-translational modification in the NSCLC cluster. (**c**) Zooming into a cluster containing expression and methylation data for the lung associated transcription factor, NKX2-1.

To demonstrate how Clustergrammer can be used for enhancing the analysis and visualization of data from various projects, several case studies are presented below. The first case study visualizes and analyzes original data collected from lung cancer cell lines. Using Tandem Mass Tag (TMT) mass spectrometry to measure differential phosphorylation, acetylation, and methylation, a panel of 42 lung cancer cell lines were compared to non-cancerous lung tissue. Corresponding gene expression data was also obtained for 37 of these cell lines from the CCLE. Using Clustergrammer, co-regulated clusters of post translational modifications (PTM) and mRNA levels in distinct lung cancer histologies were identified, and enrichment analysis was applied to investigate the biological processes involved in these lung cancer subtypes (Fig. 3, Supplementary Fig. 1). Lung cancer cell lines cluster largely according to their two types of histology: non-small cell lung cancer (NSCLC) and small cell lung cancer (SCLC) (Fig. 3a). At the second level of clustering, the cells cluster by sub-histology and mutations (Fig. 3a). Several genes and their protein products were identified to be regulated similarly across different PTM and gene expression levels (Fig. 3b,c). For instance, members of the keratin family display arginine methylation, lysine acetylation and phosphorylations that are strongly correlated with mRNA expression of the same genes/proteins, suggesting potential relationships between protein modification and mRNA levels for this family of genes. Additionally, the expression of the lung cancer associated transcription factor, NKX2-1, is highly correlated with its methylation and also clusters with several other lung- associated genes, for example, SFTA3 and SOX2, at the PTM level (Fig. 3c). Two distinct clusters of PTM and mRNA levels that are up-regulated in NSCLC or SCLC cell lines were identified and isolated for further analysis (Supplementary Fig. 1c,d). Enrichment analysis of the NSCLC cluster implicates cellular movement and adhesion related enriched terms from the gene ontology gene set library. Specifically, the terms cellular component movement, motility, migration, locomotion, adhesion and response to wound healing are enriched (Supplementary Fig. 1c). This observation broadly agrees with prior knowledge that NSCLC cells are known to form adherent monolayers, while SCLC grow in aggregates^26^. Enrichment analysis of the SCLC cluster strongly implicates neuronal functions based on the enriched gene ontology terms: neuron projection, axon guidance, and neuron morphology. Similarity, the up-regulated genes are enriched for neuronal related diseases, including: oligodendroglioma, multiple sclerosis, astrocytoma, and large cell neurocarcinoma. Neuronal related knockout mouse phenotypes are also enriched: abnormal morphology of neurons and spine and abnormal nervous system. These results agree with previous reports about neuronal characteristics of SCLC cell lines^27^ (Supplementary Fig. 1d). Overall clustergrammer is effective in quickly identifying molecular mechanisms and associations from this exciting new dataset. The corresponding interactive Jupyter Notebook for this case study can be accessed at: http://nbviewer.jupyter.org/github/MaayanLab/CST_Lung_Cancer_Viz/blob/master/notebooks/CST_Data_Viz.ipynb; Data Citation 1.

### Case study II: Visualization of CyTOF data of single cell immune response to PMA treatment

**Figure 4:**
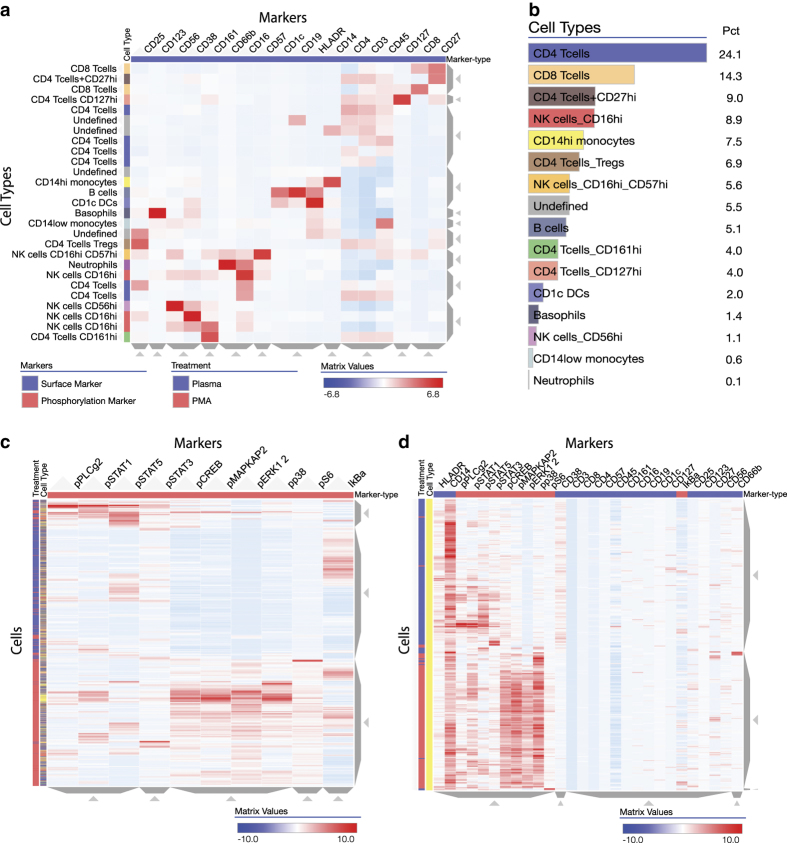
Single blood cell CyTOF data in response to PMA treatment. Single cell CyTOF data was obtained after exposing PBMCs to PMA and measuring 18 surface markers and 10 phospho-markers. (**a**) Clustergrammer was used to semi-automatically identify cell types based on surface marker expression. (**b**) Proportion of cell types based on semi-automatic identification from surface marker expression data. (**c**) Clustergrammer visualization of phospho-marker expression in single cells with cell type and treatment condition labels. (**d**) Zooming into the CD14hi monocyte cluster in phospho- and surface-marker space.

For the second case study, Clustergrammer was utilized to analyze and visualize original single-cell CyTOF data to investigate the immune response of peripheral blood mononuclear cells (PBMCs) exposed to phorbol 12-myristate 13-acetate (PMA) treatment. PMA is a known tumor promotor^28^ and an activator of protein kinase C^29^. CyTOF data was collected from over 200,000 single cells, measuring the level of 28 markers, 18 surface marker and 10 phosphorylation markers. Because of the multifaceted dimensionality, visualizing CyTOF data is difficult^30^ and sophisticated methods have been developed^31,32^. Clustergrammer was used to semi-automatically identify cell types (Fig. 4a,b) as well as to visualize cell-type specific global phosphorylation states (Fig. 4c,d). Among many observations, Clustergrammer clearly and immediately identified a unique cell-type (Fig. 4c, Supplementary Fig. 2b): PMA treated CD14hi monocytes form a cluster with high levels of pCREB, pMAPKAP2, pp38, and pERK1/2. Further investigation of this cell type in isolation reveals the that while the surface marker CD14 remained unchanged by PMA treatment, the surface markers CD38 and HLADR were downregulated after PMA exposure (Fig. 4d, Supplementary Fig. 2c). These results demonstrate that Clustergrammer can be an effective tool to analyze CyTOF data to identify rare cell types, and the cell signaling pathways that regulate these cells. The corresponding Jupyter Notebooks of this case study can be found at: http://nbviewer.jupyter.org/github/MaayanLab/Cytof_Plasma_PMA/blob/master/notebooks/Compare_Cell-Type_Distribution_PMA_Treatment.ipynb and http://nbviewer.jupyter.org/github/MaayanLab/Cytof_Plasma_PMA/blob/master/notebooks/Plasma_vs_PMA_Phosphorylation.ipynb; Data Citation 2.

### Case study III: Visualization of the cancer cell line encyclopedia gene expression data

**Figure 5:**
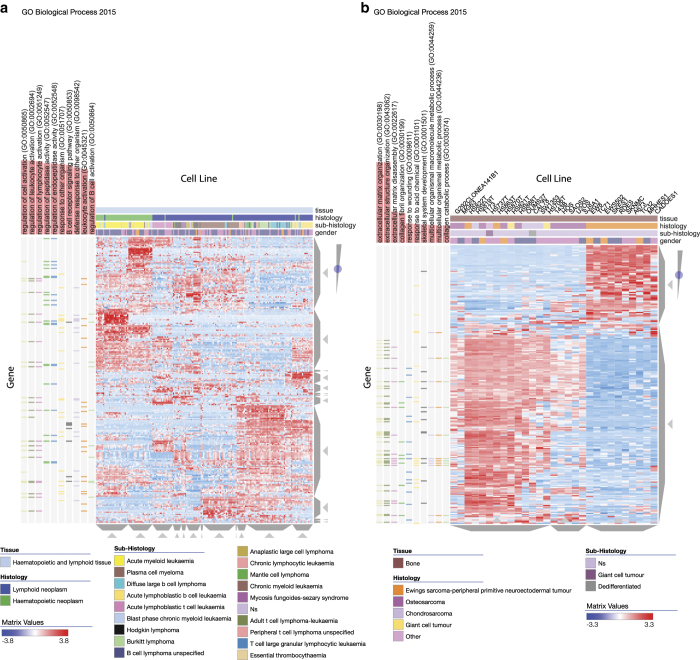
Cancer cell line encyclopedia (CCLE) gene expression data. Clustergrammer was applied to visualize the CCLE gene expression data. The CCLE Explorer available at https://maayanlab.github.io/CCLE_Clustergrammer/ allows users to explore tissue expression using heatmaps that are pre-loaded with enrichment results from the Gene Ontology Biological Process from the Enrichr library. (**a**) Haematopoietic and Lymphoid tissue cell lines (columns) heatmap with Gene Ontology Biological Process enrichment. (**b**) Bone tissue cell lines (columns) heatmap with Gene Ontology Biological Process enrichment.

The third case study involves interactive visualization of the gene expression data from the Cancer Cell Line Encyclopedia (CCLE)^33^. The Clustergrammer CCLE Explorer (Fig. 5a,b, Supplementary Fig. 3a) enables the exploration of the CCLE gene expression data by dividing the profiled cell lines into groups based on their tissue of origin, and then visualizing the top 250 most variably expressed genes within each group. Users can choose a tissue by clicking on an entry on a TreeMap view where the size of rectangles reflects the number of cell lines originating from a tissue (Supplementary Fig. 3a). Each heat map displays the histology, sub-histology, and gender of the cell line, and enrichment analysis is preloaded with enrichment results against the gene set library Gene Ontology Biological Process^34^. For instance, selecting the hematopoietic and lymphoid collection of cancer cell lines (Fig. 5a), demonstrates that these cell lines cluster by sub-histology, but such clustering is not perfect. For example, exploring the diffuse large B cell lymphoma cell lines, which are clustered within the plasma cell myeloma cluster, can potentially identify unique mechanisms and potential mislabeling. The CCLE data is also visualized within a Jupyter Notebook where specific tissues are explored in more depth, and K-means down-sampling is implemented to obtain an overview of the entire dataset (Supplementary Fig. 3b). The corresponding site for this case study can be found at: https://maayanlab.github.io/CCLE_Clustergrammer/ and the Jupyter Notebook of this case study can be found at: http://nbviewer.jupyter.org/github/MaayanLab/CCLE_Clustergrammer/blob/master/notebooks/Clustergrammer_CCLE_Notebook.ipynb; Data Citation 3.

### Additional case studies examples and documentation

**Figure 6:**
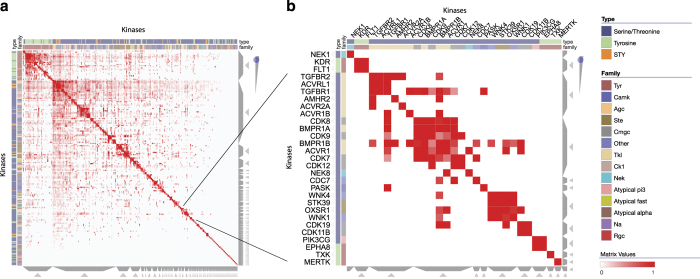
Network visualization. (**a**) Clustergrammer was used to visualize a network of kinases based on shared substrates. The network includes 404 kinases and over 100,000 kinase-kinase associations. (**b**) Zoomed view of a cluster of kinases.

Currently, Clustergrammer has been used to visualize and analyze a wide variety of data including protein-protein interaction networks (Fig. 6; Data Citation 4; https://maayanlab.github.io/kinase_substrate_similarity_network/), handwritten image data http://nbviewer.jupyter.org/github/MaayanLab/MNIST_heatmaps/blob/master/notebooks/MNIST_Notebook.ipynb#Visualize-Downsampled-Version-of-MNIST, and USDA nutritional data http://nbviewer.jupyter.org/github/MaayanLab/USDA_Nutrients_Viz/blob/master/USDA_Nutrients.ipynb. These case studies are further described in the extensive user manual and other online documentation available at: https://clustergrammer.readthedocs.io/index.html.

## Discussion

So far, as of August 2017, Clustergrammer was accessed by over 34,000 users based on Google Analytics, while integrated within several web-based applications^23,25,35–37^. Clustergrammer is modular and applicable in a wide variety of contexts. Clustergrammer is designed to be flexible in terms of the underlying computational algorithms, and this allows developers to swap in and out different algorithms with different parameters. For instance, a hybrid K-means downsampling and hierarchical clustering method was used to visualize the CyTOF single cell data (Supplementary Fig. 2b), which required additional components to be added to the core codebase. Clustergrammer is an open source project that is available on GitHub https://github.com/MaayanLab/clustergrammer enabling the opportunity for collaborative development by the broader computational biological community. Some enhancements to improve Clustergrammer can include: a) building the heatmaps using WebGL for improved performance and scalability; b) incorporating other visualization methods of the loaded data, for example, ball-and-stick network diagrams; c) enabling on-the-fly machine learning methods by dividing the uploaded data into attributes and classes, and providing a choice for setting up and running various machine learning engines.

## Methods

### Project Documentation

Documentation for the Clustergrammer project can be found at http://clustergrammer.readthedocs.io/. Clustergrammer’s documentation was built using the Python documenting tool Sphinx and is hosted by readthedocs.org. The documentation source code can be found on GitHub: https://github.com/MaayanLab/clustergrammer-docs.

### Clustergrammer core libraries

Developers can use Clustergrammer’s two core libraries Clustergrammer-JS and Clustergrammer-PY as a visualization toolkit to generate visualizations for their own projects. Clustergrammer-JS is the front-end JavaScript library that builds the interactive heatmap visualization in SVG using the JavaScript library D3.js. Clustergrammer-JS takes as input a Visualization-JSON. Clustergrammer-PY is the back-end Python library that is used to hierarchically cluster the data and generate the Visualization-JSON for the front end Clustergrammer-JS library. The Visualization-JSON contains several pre-calculated views of the data.

### Clustergrammer-JS

Clustergrammer-JS is the front end JavaScript library that builds the interactive clustergram visualization in SVG using the visualization library D3.js. The Clustergrammer-JS library is utilized by the Clustergrammer web app and the Jupyter Widget. Clustergrammer-JS takes as input the Visualization-JSON, which contains the information necessary to build the visualization. Clustergrammer-JS can be installed using the node package manager (https://www.npmjs.com/package/clustergrammer) and the open source code and can be found on GitHub (https://github.com/MaayanLab/clustergrammer).

Clustergrammer-JS is built with Webpack Module Bundler and the source code is available in the GitHub repo. Clustergrammer-JS’s API enables developers to add customized behavior through callbacks. For example, tooltip behavior can be modified using callback functions. Clustergrammer-JS utilizes the D3.js JavaScript library for data binding, where data is bound to visual elements such as SVG elements. Clustergrammer-JS also utilizes D3.js general update pattern and the visualization concept of ‘object constancy’ to enable users to track data transformations in three distinct steps: old data is removed, persistent data is updated, and new data enters the visualization. Clustergrammer is a stateful visualization that can be modified by the user, but also returned to its initial state at any time. To enable this, the Clustergrammer-JS object keeps track of the initial and current state as separate parameter objects. See http://clustergrammer.readthedocs.io/clustergrammer_js.html#clustergrammer-js-api for more information. Clustergrammer-JS was developed simultaneously with Clustergrammer-PY as new features generally required updates to both the front and back end Clustergrammer components as well as the Visualization-JSON that is exchanged between the two.

Clustergrammer-JS integrates data from external sources, for example Enrichr, by communicating with RESTful APIs that allow cross-origin-requests. Clustergrammer only activates Enrichr functionality if it identifies that row names are official mammalian Entrez gene symbols. This is achieved through Harmomizome API requests, and it is employed to prevent biologically relevant features to become activated when non-gene centric biological datasets are loaded.

### Clustergrammer-PY

Clustergrammer-PY is the back end Python library that is used to hierarchically cluster the data and generate the Visualization-JSON for the front end Clustergrammer-JS library. Clustergrammer-PY is compatible with Python 2 and 3. This library is utilized by the web app and Jupyter Widget. Clustergrammer-PY can also be used to pre-filter, normalize, down sample, and randomly sample data before the clustering is calculated. Users can modify the clustering parameters, for example setting the distance metric and linkage type, using the API. Clustergrammer-PY can be installed using pip (https://pypi.python.org/pypi?:action=display&name=clustergrammer) and the source code can be found on GitHub (https://github.com/MaayanLab/clustergrammer-py). Hierarchical clustering is calculated using the SciPy library. K-means clustering is calculated using the SciKit Learn library.

### Clustergrammer-Widget

The Clustergrammer-Widget enables users to build interactive visualizations within shareable Jupyter Notebooks. The Clustergrammer-Widget utilizes the core Clustergrammer-JS and Clustergrammer-PY libraries. Clustergrammer-Widget was built using the widget-Cookiecutter template (https://github.com/jupyter-widgets/widget-cookiecutter). Clustergrammer-Widget can be installed using pip (https://pypi.python.org/pypi/clustergrammer_widget) and the source code can be found on GitHub (https://github.com/MaayanLab/clustergrammer-widget). The Clustergrammer-Widget uses the Clustergrammer-PY API to load data, normalize, filter, calculate clustering, and finally build the interactive widget.

The Clustergrammer-Widget is implemented as a Widget class which is passed to the Clustergrammer-PY object. This widget class is based on the ipywidgets class (see https://github.com/jupyter-widgets/ipywidgets for details). It contains both the front and back end Clustergrammer core libraries (Clustergrammer-JS and Clustergrammer-PY). Widgets allow two-way communication between front and back end components, which enables users to pass data to the Python kernel from the front-end visualization (see http://clustergrammer.readthedocs.io/clustergrammer_py.html#clustergrammer_py.Network.widget_df method for an example). Widgets can be saved within Jupyter Notebooks and shared using the Nbviewer service. Nbviewer loads the widget front end using Node Package Manager.

### Clustergrammer-Web

The Clustergrammer web application enables users to easily generate shareable interactive visualizations of their data. Clustergrammer-Web is built using the Flask Python library, and is deployed as a Dockerized application. Clustergrammer-Web utilizes the core Clustergrammer-JS and Clustergrammer-PY libraries. User’s uploaded data is stored in a MongoDB database. Data is hierarchically clustered on the server side using the Python library SciPy with default parameters: cosine distance metric and average linkage type. Additional row-filtered ‘views’ of the user’s data are calculated by running successive row-filtering and re-clustering. These filtered views are available on the front-end using the row-filter sliders. The RESTful API enables developers to automatically generate visualizations. Clustergrammer can visualize matrices with as many as 500,000 cells. Clustergrammer-Web implements most of the features that are also available in the Clustergrammer-Widget and the core libraries Clustergrammer-JS/Clustergrammer-PY.

### Code availability

All code associated with the Clustergrammer project is open source. The code is available on GitHub under the MIT license. Clustergrammer libraries are versioned according to the Semantic Versioning 2.0.0 guidelines (http://semver.org/). The Clustergrammer JavaScript (front-end) library is available at https://github.com/MaayanLab/clustergrammer. The Clustergrammer Python library is available at https://github.com/MaayanLab/clustergrammer-py. The Clustergrammer Jupyter Widget is available at https://github.com/MaayanLab/clustergrammer-widget. The Clustergrammer web application is available at https://github.com/MaayanLab/clustergrammer-web. See online documentation (http://clustergrammer.readthedocs.io/) for more information about code availability and contributing to the Clustergrammer project.

### Tandem mass tag (TMT) experiments to determine PTMs in lung cancer cell lines

PTMs of 45 lung cancer cell lines, 12 derived from SCLC and 33 from NSCLC, were compared to normal lung tissue pooled from anonymous patients using an established protocol^38^. Briefly, cells were washed and harvested in PBS and cell pellets frozen in liquid nitrogen. Cells were lysed in a 10:1 (vol/wt) volume of lysis buffer (4% SDS; 100 mM NaCl; 20 mM HEPES pH 8.5, 5 mM DTT, 2.5 mM sodium pyrophosphate; 1 mM β-glycerophosphate; 1 mM Na3VO4; 1 μg ml^−1^ leupeptin), and proteins were reduced at 60 °C for 45 min. Proteins were then alkylated by the addition of 10 mM iodoactamide (Sigma) for 15 min at room temperature in the dark, and methanol/chloroform precipitated. Protein pellets were resuspended in urea lysis buffer (8 M urea; 20 mM HEPES pH 8.0; 1 mM sodium orthovanadate; 2.5 mM sodium pyrophosphate; 1 mM β-glycerolphosphate) and sonicated. Insoluble material was removed by centrifugation 10,000×*g*, 5 min, and the supernatant diluted fourfold in 20 mM HEPES pH 8.5, 1 mM CaCl2, for Lys-C digestion overnight at 37 °C, then diluted two-fold and trypsin digestion 4–6 h at 37 °C. Samples were then acidified to pH 2–3 with formic acid, peptides purified on a Waters Sep-Pak column and dried in a speed-vac. Peptides were purified on a Waters Sep-Pak column, and quantified using a micro-BCA assay (Thermo). Mass tag (6-plex TMT reagents; Thermo) were crosslinked to peptides in 30% acetonitrile/200 mM HEPES pH 8.5 1 h at room temperature and the reaction stopped by the addition of 0.3% (v/v) hydroxyamine. Samples are then mixed in equimolar ratios, and the ratios checked and samples run on an Orbitrap Exactive MS (Thermo). Combined samples were then sequentially immunoprecipitated with cocktails of modification-specific antibodies in the order: anti-phosphotyrosine; anti-phosphoserine/threonine; anti-methylarginine; anti-metyllysine; and anti-acetyllysine (Cell Signaling Technology). After anti-phosphotyrosine and anti-phosphoserine/threonine immunoprecipitation phosphopeptides were further purified on a TiO2 column (Thermo). Identification of peptides and quantification of mass tags was obtained from the the MS2 spectrum after fragmentation by MS/MS analysis. In order to compare this PTM data to gene expression data from the CCLE, only cell lines that were included in both datasets (37 out of 42 cell lines) were included. PTM ratios for each lung cancer cell line were calculated by dividing PTM levels in each lung cancer cell line by non-cancerous lung tissue PTM levels in the corresponding multiplex run. PTM ratio levels were quantile normalized in each cell line to make the distributions comparable. PTMs with more than seven missing values were removed to reduce the global effects of the missing data. Finally, PTM levels were Z-score normalized to emphasize relative changes across cell lines. Gene expression data for the 37 assayed lung cancer cell line was obtained from CCLE. The top 1,000 genes with the highest variance across these cell lines were kept for further processing. Finally, genes were Z-scored across lung cancer cell lines to emphasize relative changes across cell lines. Interactive clustergrams were produced from the PTM data (1,730 PTMs×37 cell lines), gene expression data (1,000 genes×37 cell lines), and combined PTM-expression data (2,730 PTMs/genes×37 cell lines) with the default hierarchical clustering parameters cosine distance and average linkage. Up-regulated clusters of PTMs/genes in NSCLC and SCLC clusters were exported from the visualization for further processing using the Clustergrammer-PY’s *widget_df* method. Supporting code can be found at https://github.com/MaayanLab/CST_Lung_Cancer_Viz and the Jupyter notebook http://nbviewer.jupyter.org/github/MaayanLab/CST_Lung_Cancer_Viz/blob/master/notebooks/CST_Data_Viz.ipynb.

### CyTOF data single cell immune response to PMA treatment

CyTOF (Fluidgm) was utilized by the Icahn School of Medicine Human Immune Monitoring Core investigate the phosphorylation-level response of single PBMCs exposed to PMA. CyTOF data was pre-processed to remove cell doublets and converted to comply to Clustergrammer’s input data format. Equal sized subsets (110,000 single cells) were taken from the PMA and non-treated conditions to construct a dataset with 220,000 single cells (rows) and 28 markers (columns). To obtain overviews of the dataset the data was first Z-score normalized along the columns and then either randomly subsampled or K-means down sampled. Down sampling was performed using K-means clustering to obtain 2,000 clusters from the 220,000 single cells. Each K-means cluster has a Majority-Treatment category flag: Plasma (untreated) or PMA. The size of each cluster is also indicated in the visualizations with the ‘number in clust’ value-based category (the second row category) and cluster sizes range from 2 to ~450 cells. Cell types were semi-automatically identified using hierarchical clustering of down sampled cell data in surface-marker space. Single cell data in surface-marker space were down sampled to 2,000 K-means clusters and hierarchically clustered. 27 cell-clusters were manually identified based on surface marker expression. These manually assigned labels were transferred back to the single cell level for further processing. Cells were visualized in phosphorylation space using random subsampling (2,000 cells were randomly chosen from PMA and Plasma treatments) and K-means downsampling. Supporting code can be found at https://github.com/MaayanLab/Cytof_Plasma_PMA and the Jupyter notebook http://nbviewer.jupyter.org/github/MaayanLab/Cytof_Plasma_PMA/blob/master/notebooks/Plasma_vs_PMA_Phosphorylation.ipynb.

### CCLE gene expression data visualization

The CCLE gene expression data was obtained from the Broad Institute’s website at https://software.broadinstitute.org/software/cprg/?q=node/11. The data was pre-processed using the process_CCLE.py script to integrate cell line meta-data into a matrix. The Jupyter Notebook Calculate_CCLE_Tissue_Heatmaps.ipynb was used to calculate tissue-of-origin clustergrams. This notebook gathers all cell lines from a particular tissue of origin, filters for the top 250 most variable genes using Clustergrammer-PY, Z-score normalizes genes across all cell lines in the group, pre-calculates enrichment analysis results for Gene Ontology Biological Process using Clustergrammer-PY, calculates hierarchical clustering, and saves the Visualization-JSONs for each tissue. The tissue treemap for the CCLE Explorer was generated using D3.js and the page is hosted on GitHub. The Jupyter notebook Clustergrammer_CCLE_Notebook.ipynb investigates several tissues and generates a global overview of the entire dataset. The notebook generates a global overview of the CCLE gene expression data using K-means down sampling implemented in Clustergrammer-PY. Supporting code can be found at https://github.com/MaayanLab/CCLE_Clustergrammer and the Jupyter notebook http://nbviewer.jupyter.org/github/MaayanLab/CCLE_Clustergrammer/blob/master/notebooks/Clustergrammer_CCLE_Notebook.ipynb.

## Additional Information

**How to cite this article:** Fernandez, N. F. *et al.* Clustergrammer, a web-based heatmap visualization and analysis tool for high-dimensional biological data. *Sci. Data* 4:170151 doi: 10.1038/sdata.2017.151 (2017).

**Publisher’s note:** Springer Nature remains neutral with regard to jurisdictional claims in published maps and institutional affiliations.

## Supplementary Material

Supplementary Information
