# Supplementary material for: Clustergrammer, a web-based heatmap visualization and analysis tool for high-dimensional biological data
Source: Sci Data. 2017 Oct 10;4:170151. doi: 10.1038/sdata.2017.151 (PMC5634325; doi:10.1038/sdata.2017.151)
Supplement: Supplementary Information [file sdata2017151-s1.pdf]

Supplementary Information for:

Clustergrammer, a web-based heatmap visualization and analysis tool for high-dimensional biological data

Nicolas F. Fernandez[1], Gregory W. Gundersen[1], Adeeb Rahman[2], Mark L. Grimes[3], Klarisa Rikova[4], Peter Hornbeck[4], Avi Ma'ayan[1,*]

[1]Department of Pharmacological Sciences, Mount Sinai Center for Bioinformatics, BD2K-LINCS Data Coordination and Integration Center (DCIC), Icahn School of Medicine at Mount Sinai, New York, NY 10029, USA

[2]Human Immune Monitoring Core, Icahn School of Medicine at Mount Sinai, New York, NY 10029, USA

[3]Center for Structural and Functional Neuroscience, University of Montana, Missoula, MT 59812, USA

[4]Cell Signaling Technology Inc., Danvers, MA 01923, USA

[*]To who correspondence should be addressed: avi.maayan@mssm.edu

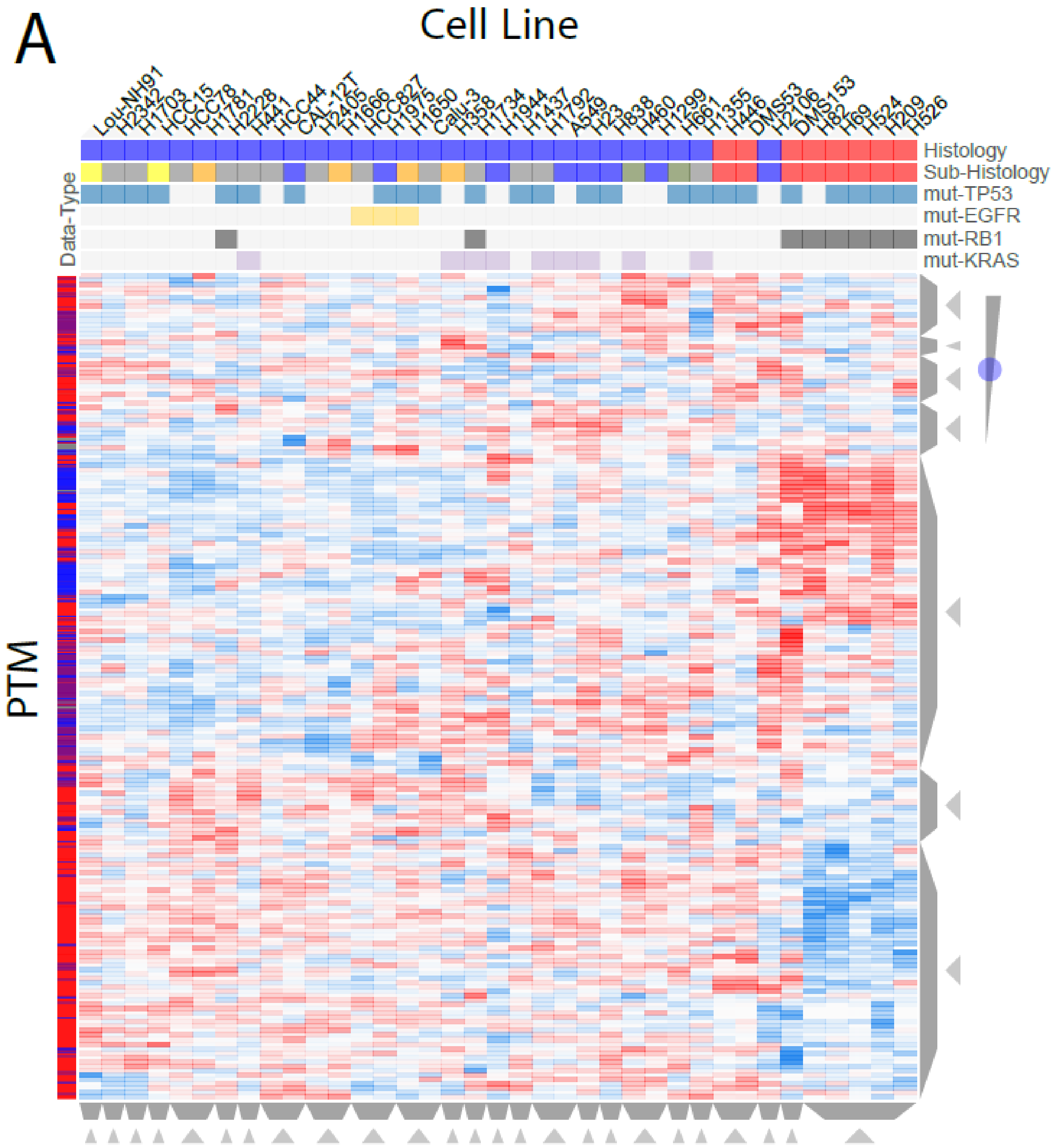
A
Cell Line
Lou-NH91
H2342
H1703
HCC15
HCC78
H1781
H2228
H441
HCC44
CAL-12T
H2405
H1666
HCC827
H1975
H1650
Calu-3
H358
H1734
H1944
H1437
H1792
A549
H23
H838
H460
H1299
H661
H1355
H446
DMS53
H2106
DMS153
H82
H69
H524
H209
H526
Histology
Sub-Histology
mut-TP53
mut-EGFR
mut-RB1
mut-KRAS
Data-Type
PTM

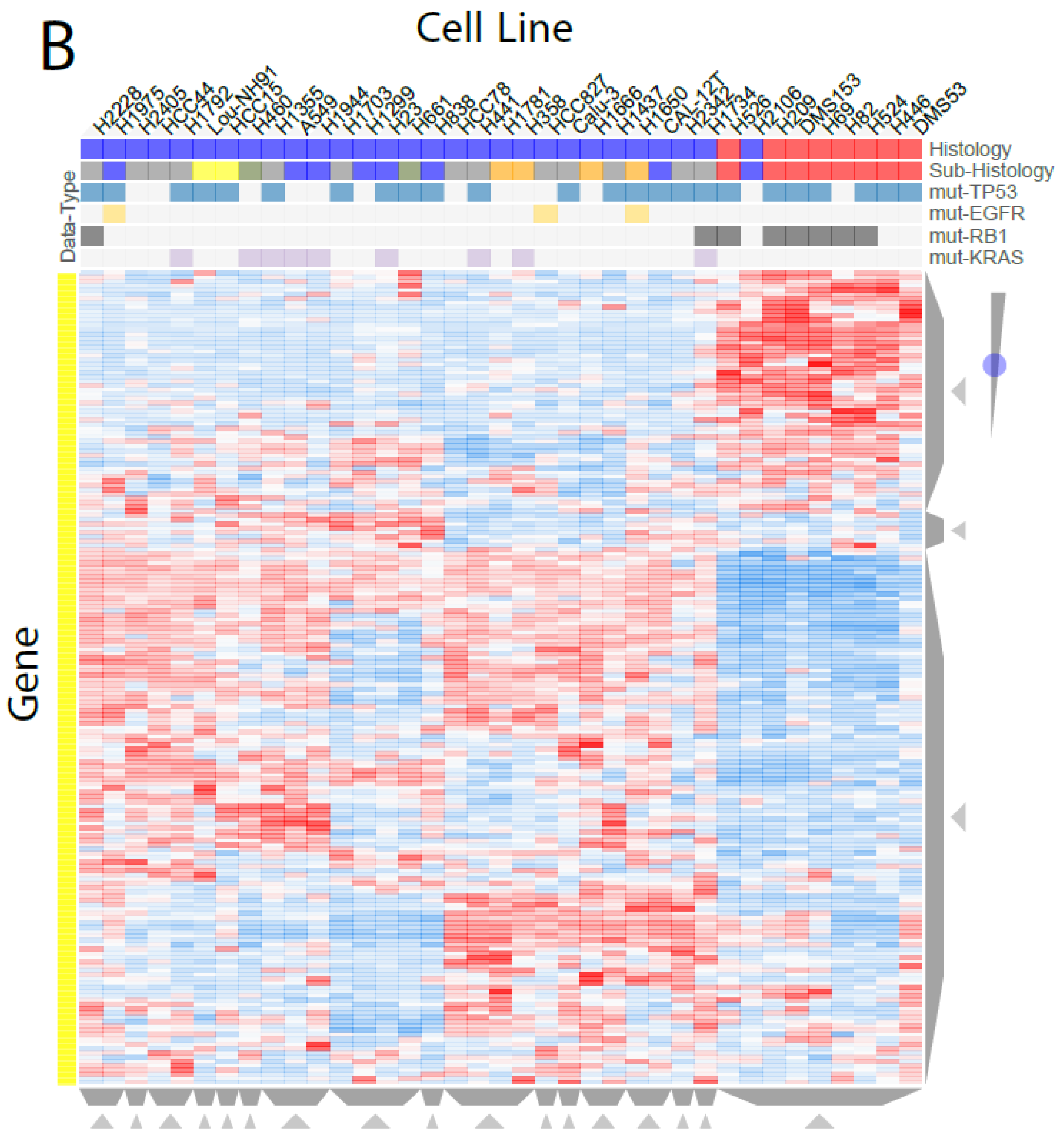

B
Cell Line
H2228
H1975
H2405
HCC44
H1792
Lou-NH91
HCC15
H460
H1355
A549
H1944
H1703
H1299
H23
H661
H838
HCC78
H441
H1781
H358
HCC827
Calu-3
H1666
H1437
H1650
CAL-12T
H2342
H1734
H526
H2106
H209
DMS153
H69
H82
H524
H446
DMS53
Data-Type
Histology
Sub-Histology
mut-TP53
mut-EGFR
mut-RB1
mut-KRAS
Gene

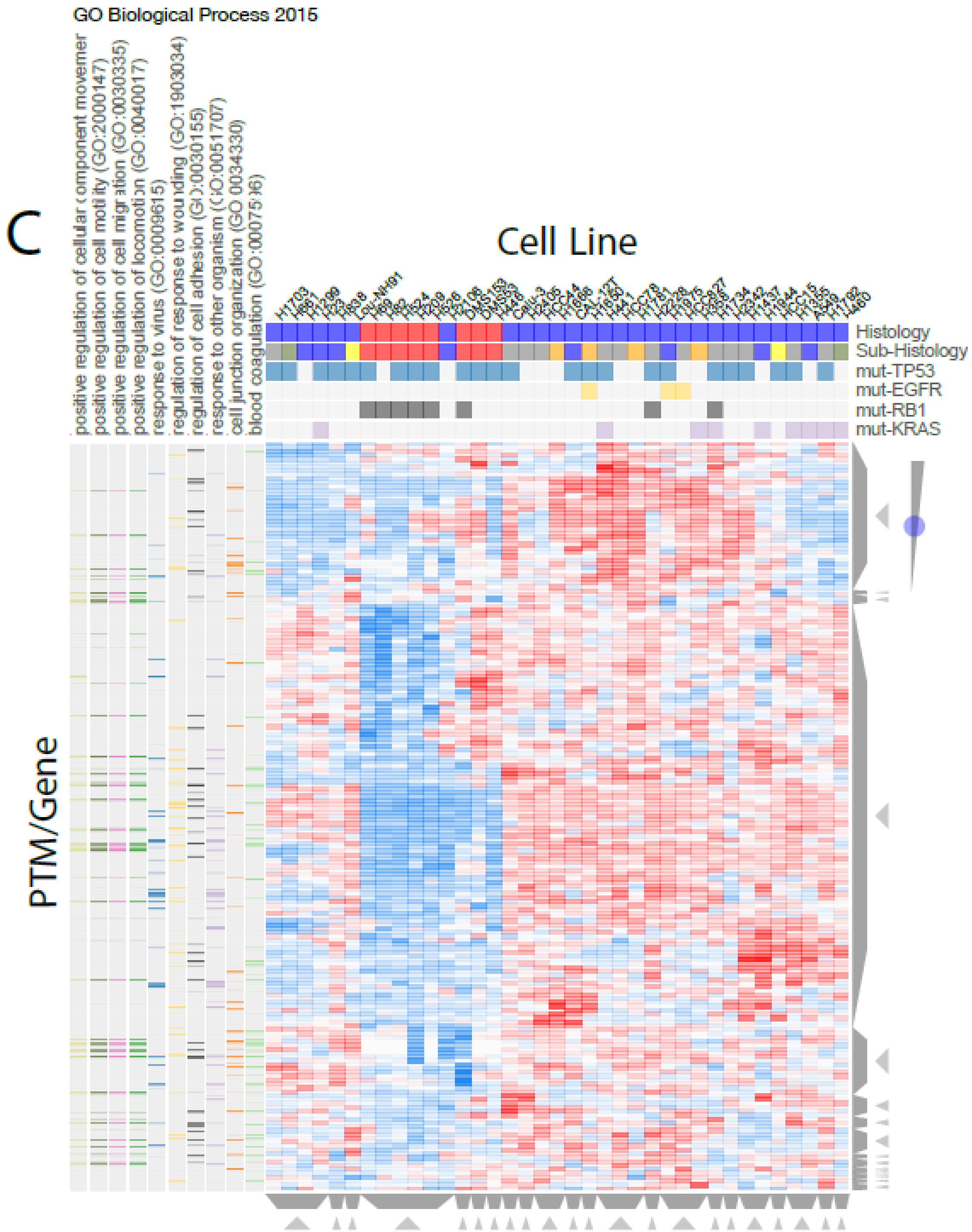
GO Biological Process 2015
C
Cell Line
PTM/Gene
Histology
Sub-Histology
mut-TP53
mut-EGFR
mut-RB1
mut-KRAS
positive regulation of cell motility (GO:2000147)
positive regulation of cell migration (GO:0030335)
positive regulation of locomotion (GO:0040017)
response to virus (GO:0009615)
regulation of response to wounding (GO:1903034)
response to other organism (GO:0051707)
cell junction organization (GO:0034330)

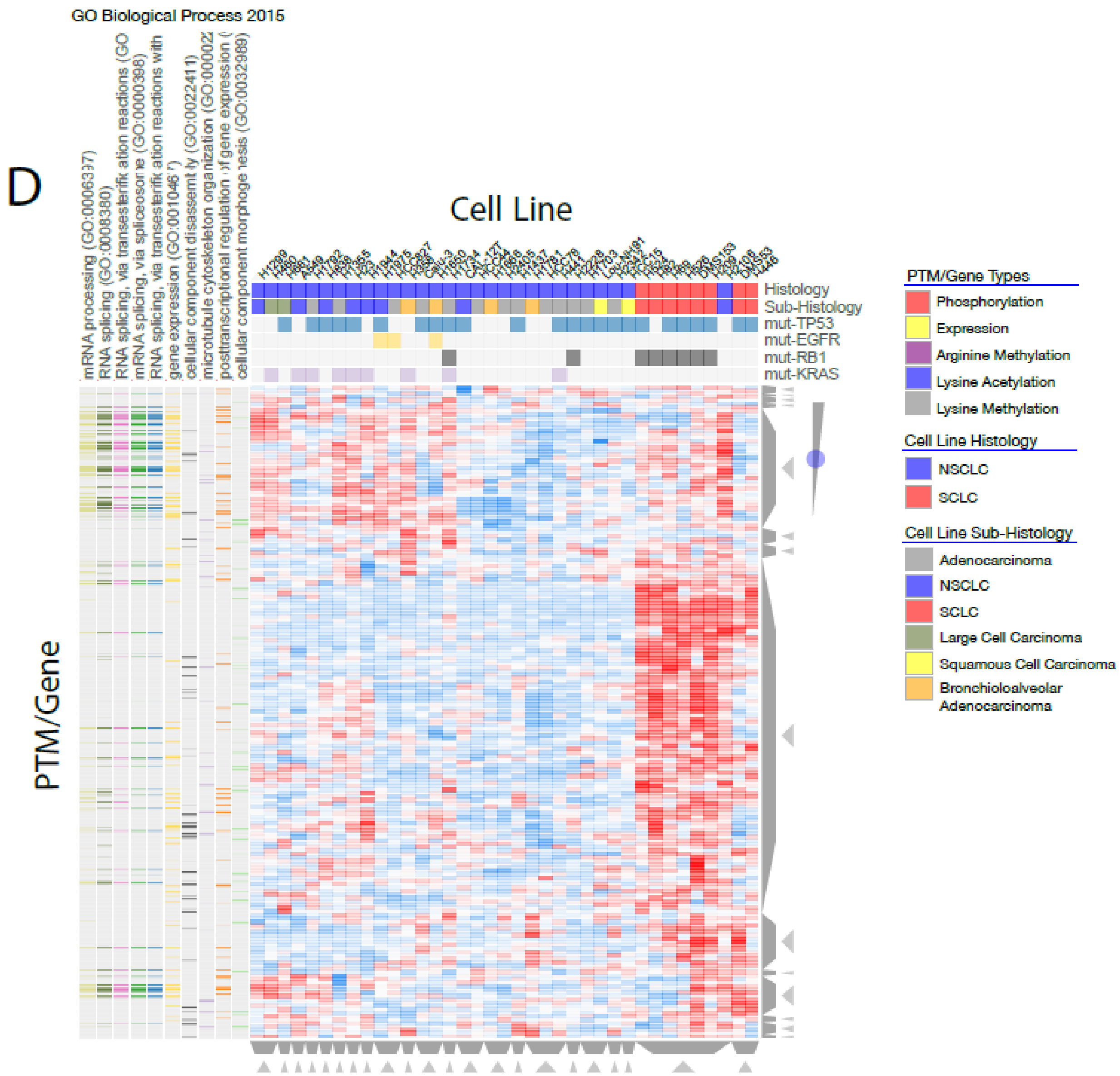

Supplementary Fig. 1. Lung cancer post-translational modifications and gene expression regulation. (a) Lung cancer cell lines (columns) were clustered based on PTM data, and (b) gene expression data. Data type is indicated using row categories. (c) Enrichment analysis of the NSCLC up-regulated PTM/gene cluster using the Gene Ontology Biological library. (d) Enrichment analysis of the SCLC up-regulated PTM/gene cluster using the Gene Ontology Biological library.

A

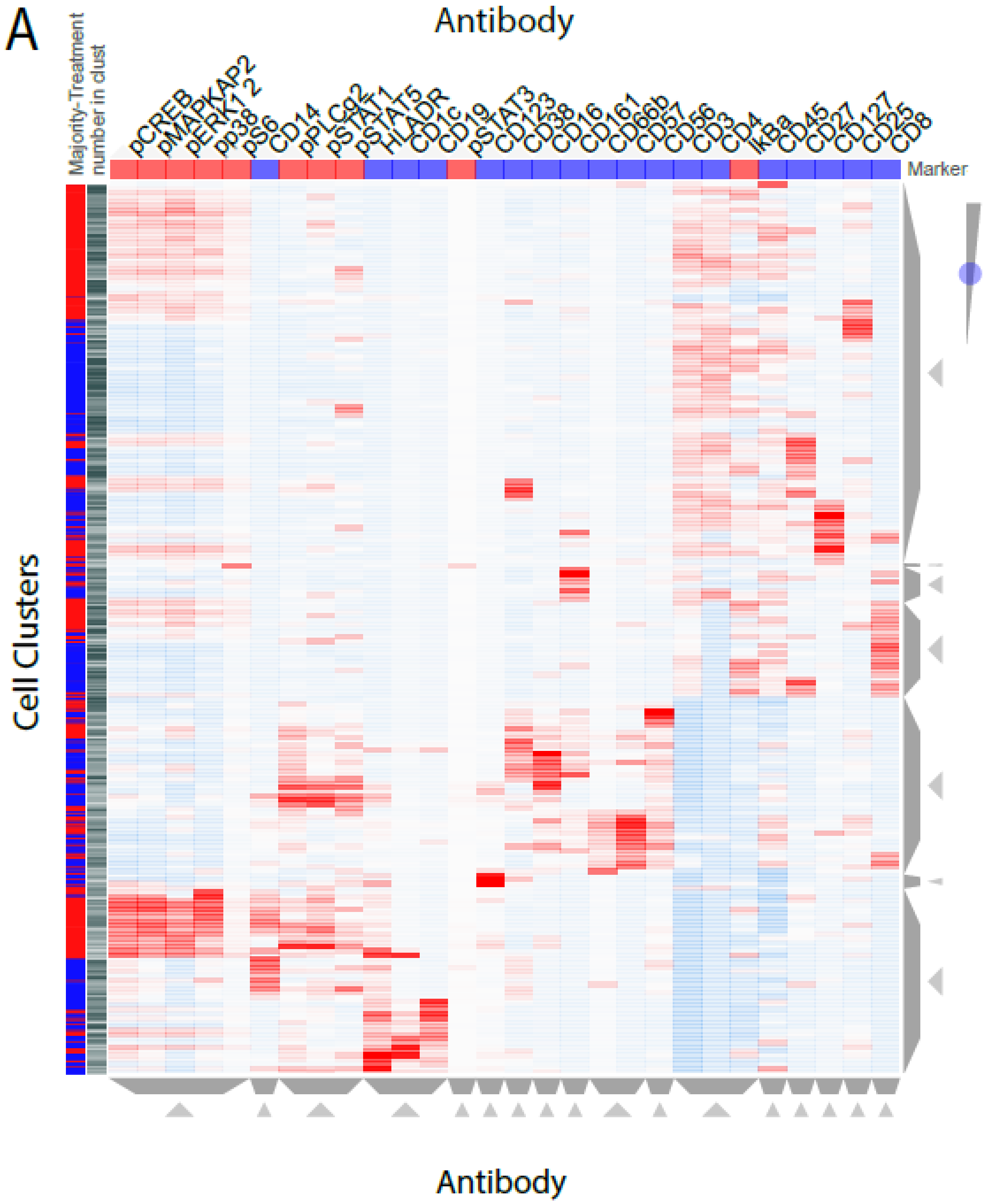

Antibody
Majority-Treatment
number in clust
pCREB
pMAPKAP2
pERK1 2
pp38
pS6
CD14
pPLCg2
pSTAT1
pSTAT5
HLADR
CD1c
CD19
pSTAT3
CD123
CD38
CD16
CD161
CD66b
CD57
CD56
CD3
CD4
IkBa
CD45
CD27
CD127
CD25
CD8
Marker
Cell Clusters
Antibody

B

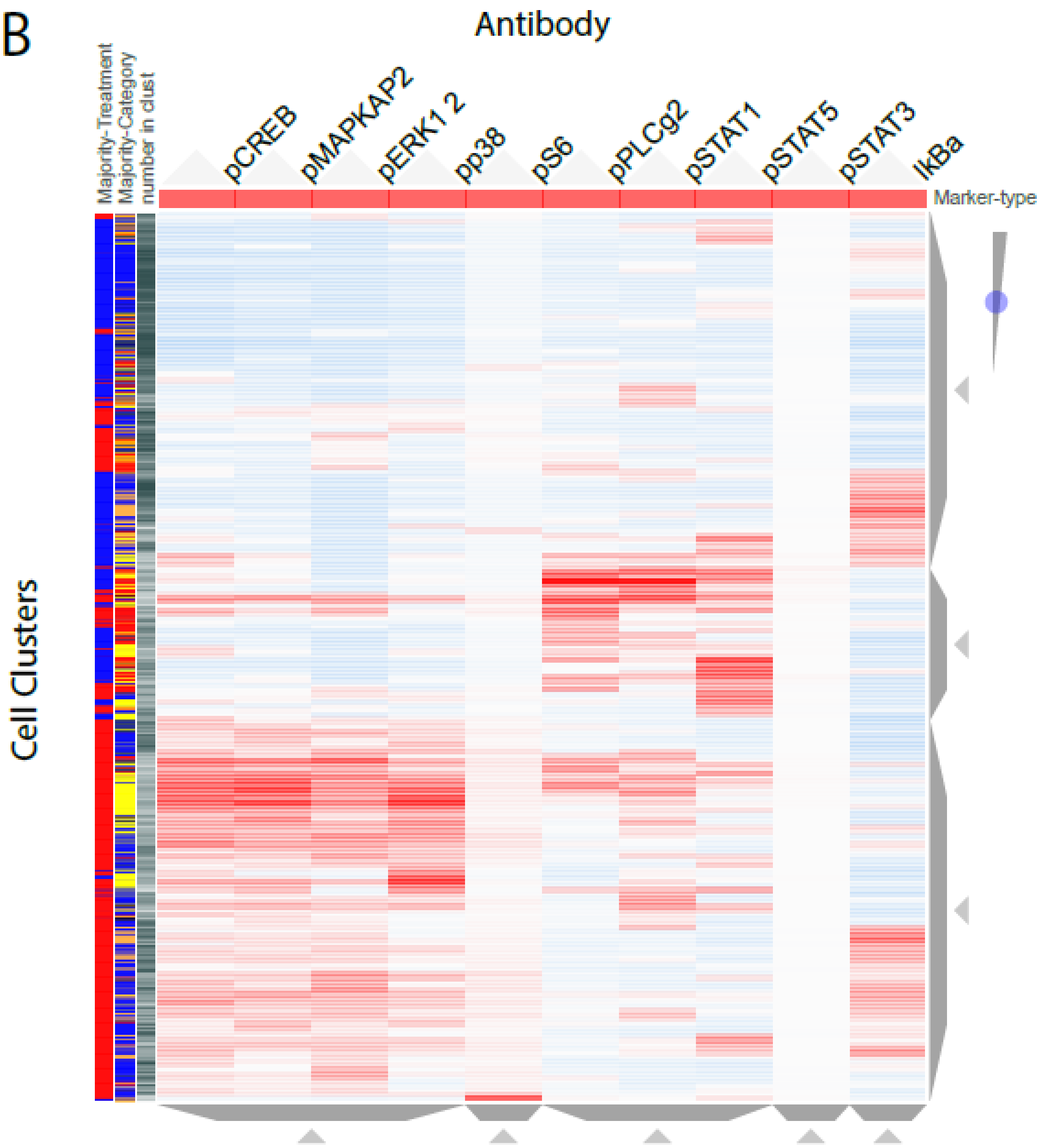
Antibody
Majority-Treatment
Majority-Category
number in clust
pCREB
pMAPKAP2
pERK1 2
pp38
pS6
pPLCg2
pSTAT1
pSTAT5
pSTAT3
IkBa
Marker-type
Cell Clusters

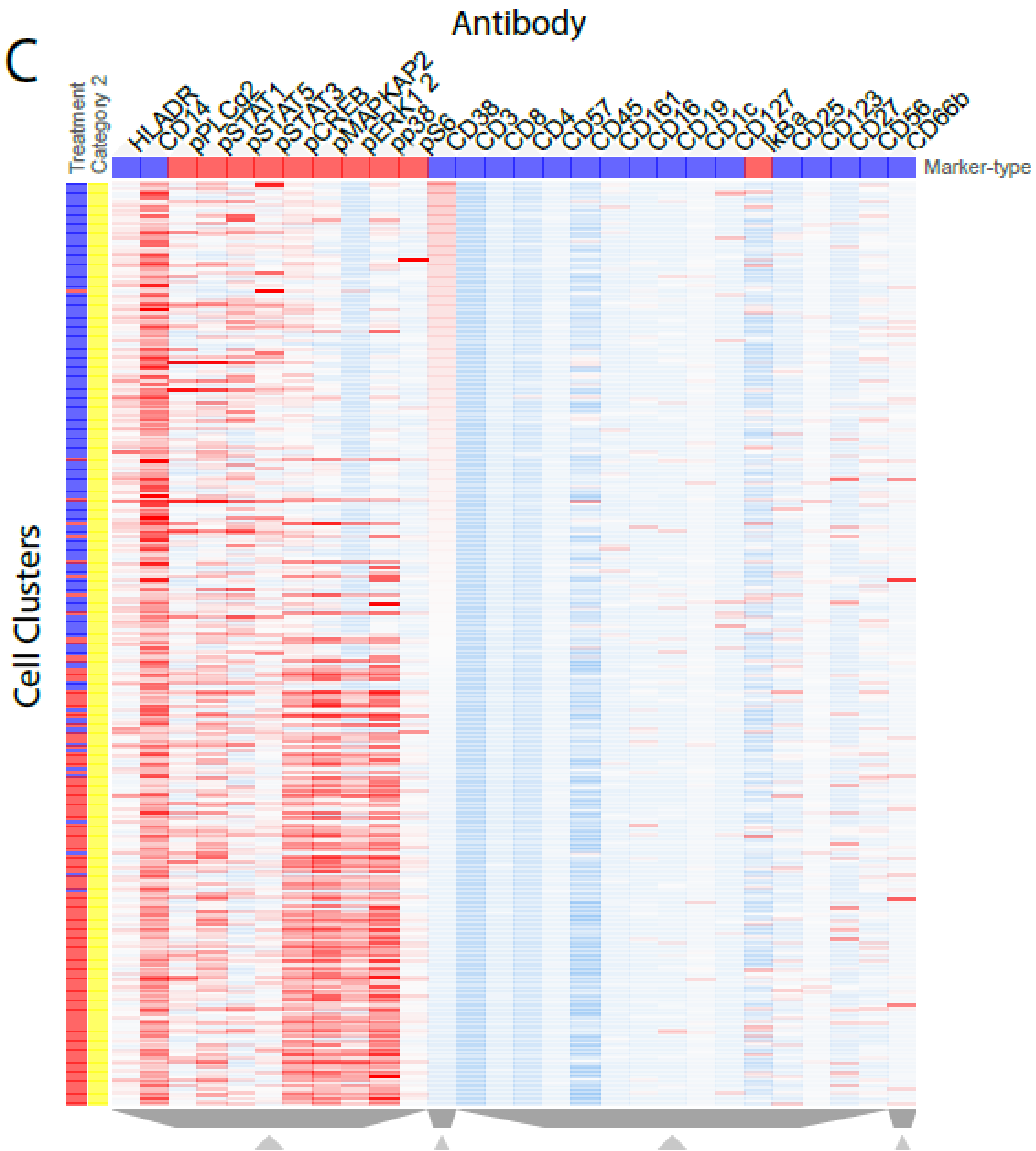

Antibody
C
Treatment
Category 2
HLADR
CD14
pPLCg2
pSTAT1
pSTAT5
pSTAT3
pCREB
pMAPKAP2
pERK1 2
pp38
pS6
CD38
CD3
CD8
CD4
CD57
CD45
CD161
CD16
CD19
CD1c
CD127
IkBa
CD25
CD123
CD27
CD56
CD66b
Marker-type
Cell Clusters

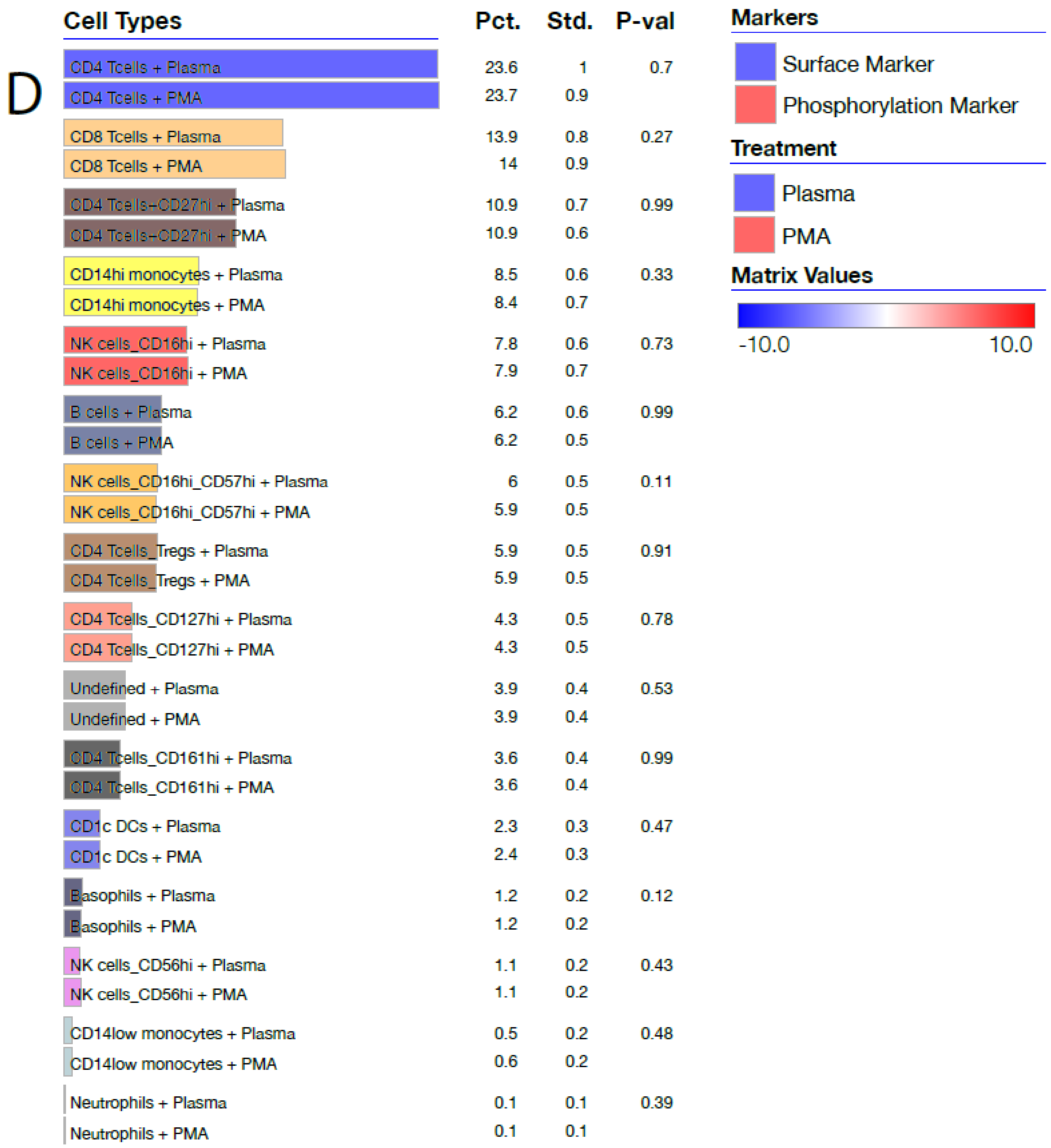

Supplementary Fig. 2. Single blood cells response to PMA treatment CyTOF Data. (a) Single cell CyTOF data was obtained after exposing PBMCs to PMA. Data is visualized using a clustergram with markers as columns (surface markers: red, phospho-markers: blue) and cells as rows (PMA treated: red, non-treated: blue). (b) K-means downsampling was used to obtain an overview of the cells in phosphorylation space after cell-type labeling (110,000 PMA and 110,000 non-treated single cells). Cell-clusters are shown as rows (clusters are defined using K-means downsampling and the cluster size is indicated using the third row category) and the majority cell-type is shown as the second row category. (c) Reordering CD14hi monocytes (isolated from subsampling) based on the surface marker CD38 level almost completely separates PMA treated cells from Plasma treated. (d) Cell population distribution with or without exposure to PMA.

A

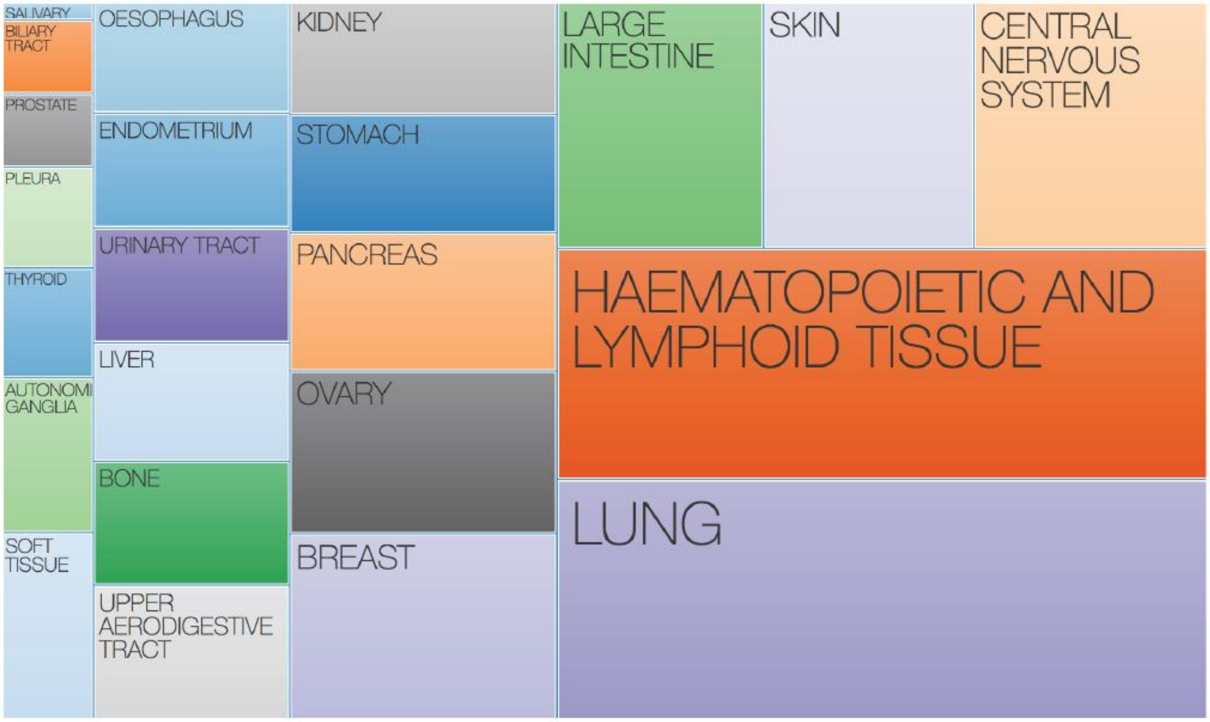
SALIVARY
BILIARY TRACT
PROSTATE
PLEURA
THYROID
AUTONOMI GANGLIA
SOFT TISSUE
OESOPHAGUS
ENDOMETRIUM
URINARY TRACT
LIVER
BONE
UPPER AERODIGESTIVE TRACT
KIDNEY
STOMACH
PANCREAS
OVARY
BREAST
LARGE INTESTINE
SKIN
CENTRAL NERVOUS SYSTEM
HAEMATOPOIETIC AND LYMPHOID TISSUE
LUNG

B

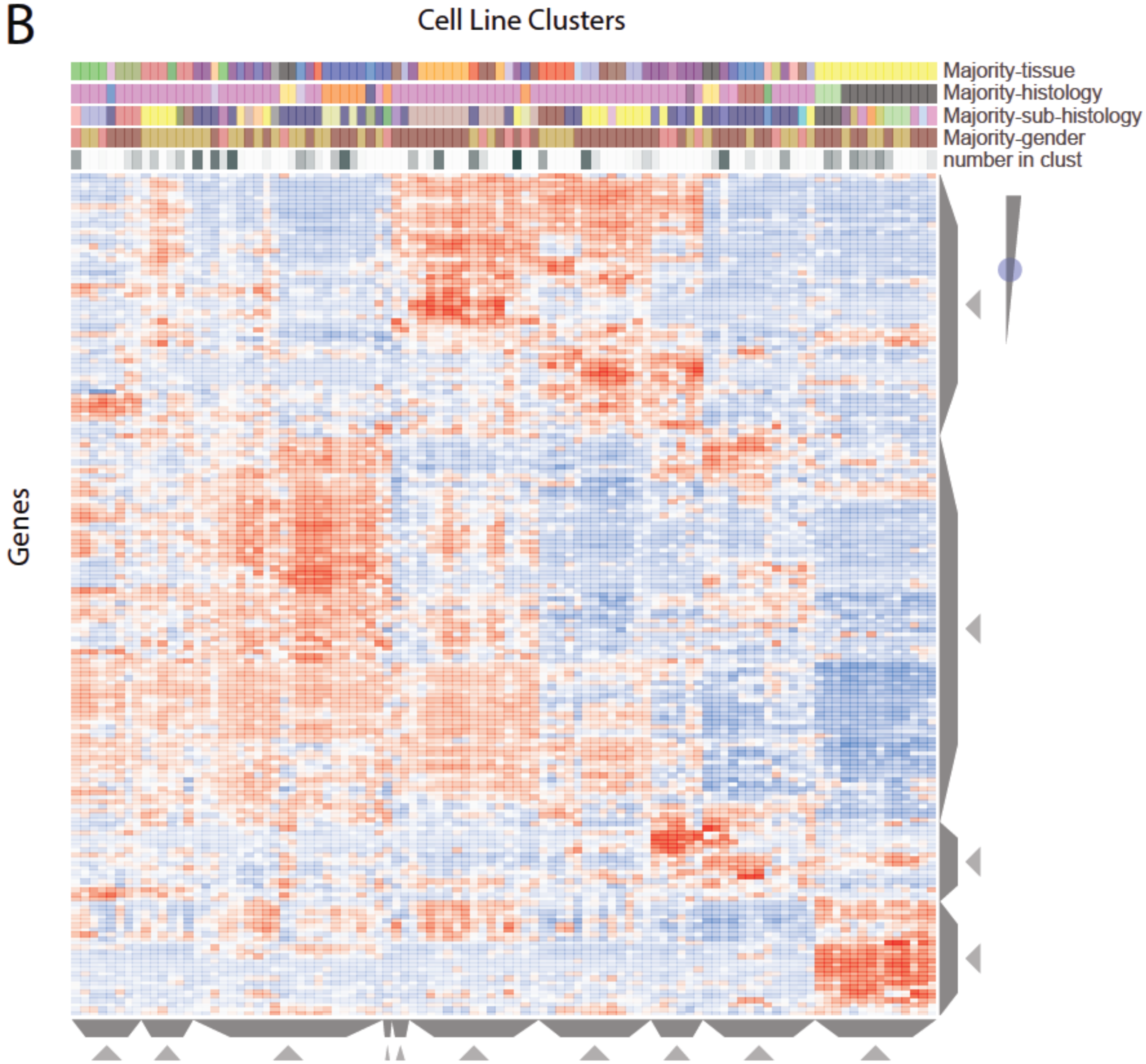
Cell Line Clusters
Majority-tissue
Majority-histology
Majority-sub-histology
Majority-gender
number in clust
Genes

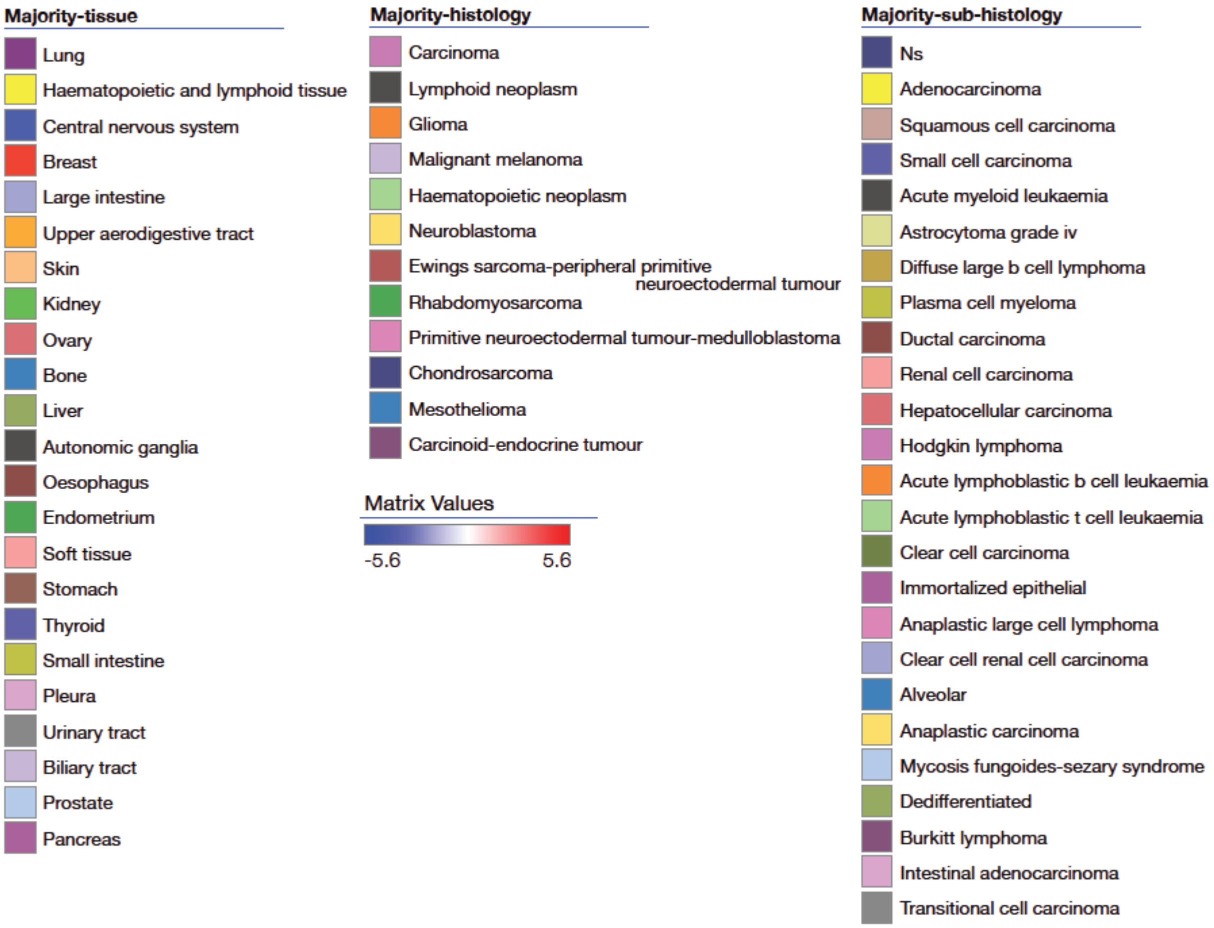

Supplementary Fig. 3. Cancer cell line encyclopedia (CCLE) gene expression data. (a) The CCLE Explorer available at https://maayanlab.github.io/CCLE_Clustergrammer/ allows users to explore tissue expression using a TreeMap visualization where the size of a tissue reflects the number of cell lines originating from a tissue. Clicking on a tissue tile displays a heatmap of the top 250 most variably expressed genes within the tissue after Z-score normalization across the rows (genes) for the cell lines within the tissue. (b) K-means downsampling to generate 100 cell line clusters, and filtering to keep the top 1000 variable genes, was implemented to obtain a visualization of the entire CCLE dataset.
